# Preclinical Models of Pediatric Brain Tumors—Forging Ahead

**DOI:** 10.3390/bioengineering5040081

**Published:** 2018-10-02

**Authors:** Tara H.W. Dobson, Vidya Gopalakrishnan

**Affiliations:** 1 Department of Pediatrics, University of Texas, M.D. Anderson Cancer Center, Houston, TX 77030, USA; 2 Department of Molecular & Cellular Oncology, University of Texas, M.D. Anderson Cancer Center, Houston, TX 77030, USA; vgopalak@mdanderson.org; 3 Brain Tumor Center, University of Texas, M.D. Anderson Cancer Center, Houston, TX 77030, USA; 4 Center for Cancer Epigenetics, University of Texas, M.D. Anderson Cancer Center, Houston, TX 77030, USA; 5 Graduate School of Biomedical Sciences UT-Health Science Center, Houston, TX 77030, USA

**Keywords:** preclinical, in vitro models, animal models, pediatric brain tumors

## Abstract

Approximately five out of 100,000 children from 0 to 19 years old are diagnosed with a brain tumor. These children are treated with medication designed for adults that are highly toxic to a developing brain. Those that survive are at high risk for a lifetime of limited physical, psychological, and cognitive abilities. Despite much effort, not one drug exists that was designed specifically for pediatric patients. Stagnant government funding and the lack of economic incentives for the pharmaceutical industry greatly limits preclinical research and the development of clinically applicable pediatric brain tumor models. As more data are collected, the recognition of disease sub-groups based on molecular heterogeneity increases the need for designing specific models suitable for predictive drug screening. To overcome these challenges, preclinical approaches will need continual enhancement. In this review, we examine the advantages and shortcomings of in vitro and in vivo preclinical pediatric brain tumor models and explore potential solutions based on past, present, and future strategies for improving their clinical relevancy.

## 1. Introduction

Preclinical models contributed to significant advancements in the field of oncology during the past century, particularly the mechanistic understanding of tumor initiation and progression, resulting in improved diagnostic and prognostic assessment of patients. However, studies to identify novel therapeutics has limited value in the clinic. Mastering the design of models that efficiently progress preclinical studies from basic science and target identification, to translational research and drug discovery, to Food and Drug Administration (FDA)-approved drugs with clinical benefit is an ongoing endeavor. Only one in 5000 compounds go on to receive FDA approval. The success rate of new cancer therapies in phase I clinical trials is a dismal 3.4%, the lowest among major diseases [1]. Novel cancer therapeutics that make it to the clinic only have a one in ten chance of helping patients, and even then, the maximum increase in survival is 5.8 months [2,3]. To date, not one of these drugs was developed specifically for pediatric patients, despite the undeniable understanding that pediatric tumors are not the same as adult cancers. Not only are the tumors diverse, but there is the additional factor of treating a growing child—for children with central nervous system (CNS) tumors, we are talking about a developing brain. For now, pediatric cancer researchers are limited to studies with repurposed compounds. Therefore, attention should be placed on designing the best models possible to accurately predict which therapeutics (1) can target heterogeneous human tumors, (2) have minimal toxic effects on a developing child, and, for brain cancers, (3) has the ability to cross the blood–brain barrier. We anticipate this will require comprehensive knowledge of past observations, benefits and limitations of current studies, and innovative ideas with the potential to move pediatric research forward. 

## 2. History of Preclinical Animal Models of Pediatric Brain Cancer

Modeling cancers has been around since 1915 when the first mice were exposed to coal tar in an attempt to create skin tumors in a laboratory [4]. It was not until 1939 when Seligman and Shear successfully utilized a carcinogen, methylcholanthrene, to generate a model for studying brain cancer [5]. Methylcholanthrene pellets were intracranially implanted into 20 mice, resulting in 11 gliomas and two meningeal fibrosarcomas [5]. They used this model for growth studies and were able to subcutaneously passage the tumors [4]. By the 1960s, with a greater understanding of carcinogenesis, studies of synthetic chemicals as possible mutagens commenced [4]. The synthetic carcinogen, *N*-nitrosourea, as well as derivatives ethylnitrosourea (ENU) and *N*-nitrosomethylurea, was found to induce brain tumors in rats [4,6]. Initial studies determined younger animals were more susceptible to tumor development compared to older rats after ENU injections. Transplacental oncogenic properties were identified after pups born from rats injected with ENU during pregnancy developed tumors [6]. An additional study, seven years later, reported a single intravenous injection of ENU at gestational day 20 resulted in tumors in 100% of the 25 offspring [7]. An astonishing total of 102 neural tumors developed in these pups consisting of oligodendrogliomas, astrocytomas, mixed gliomas, anaplastic gliomas, ependymomas, one meningioma, and neurinomas [7]. In 1936, intracerebral inoculation of RNA Rous sarcoma virus (RSV)-1, an avian retrovirus, induced intracerebral sarcoma in adult chickens [8]. By 1964, Rabotti et al. demonstrated the importance of tumor location after discovering intracerebral inoculations of RSV injected into different areas of hamster brains resulted in different tumor types [9]. A study in beagles using RSV demonstrated 100% penetrance in newborn pups after intracerebral injections. The developed tumors were shown to respond to chemotherapies from the time, (BCNU (carmustine) and CCNU (lomustine), but not cyclophosphamide [10]. These results mimicked observations in the clinic with human brain tumor patients [4]. Together with other studies, they demonstrated variations in dosage, age, strain, and overall health of the animal effected tumor type, malignancy characteristics, and location and rate of incidence [11]. In addition, this research contributed to important discoveries including the identification and characterization of blood–brain barrier defects, as well as aberrant endothelial and perivascular spread [4,12,13].

The development of brain tumor models most commonly used today began over thirty years ago. At this time, the first models of pediatric brain tumors were generated. In 1983, Neely et al. attempted to transplant 85 different pediatric tumors into immunodeficient animals, but had varying degrees of success [14]. Establishing tumors from brain in nude mice went very poorly, as did tissue from lymphoid benign tumors [14]. Simultaneously, cell lines derived from pediatric brain tumors including medulloblastoma (MB), atypical teratoid rhabdoid tumor (AT/RT), and high-grade gliomas (HGG) were developed, and, by the late 1980s, transplantable models were successfully established [15,16,17,18,19,20,21]. As the ability to genetically manipulate the mouse genome improved, mice with altered oncogenes and tumor suppressor genes were engineered [22,23,24,25]. These early patient-derived xenografts (PDX) and genetically engineered mouse models (GEMM) gained promise for use in biomedical analysis as they resembled human tumors and responded to therapeutics similar to clinical observations [4,12,13]. 

Historically, the initial inspiration to generate animal models was to test the effects of treatment on the tumors, but these older versions quickly proved to be unreliable. Tumor type and location lacked consistency with the chemically induced models [4]. By the same token, using viruses to generate brain tumors resulted in inconsistent tumor growth characteristics due to uneven distribution of recombinant viruses [4,12,13]. However, this pioneering research was instrumental for the characterization of CNS tumorigenesis, and greatly contributed to our understanding of the importance of tumor location, the surrounding microenvironment, and cells of origin [4,12,13]. This basic knowledge inspired many molecular and genetic studies of neural tumors that, in turn, resulted in improved diagnostics such as risk stratification, epidemiologic studies, and therapeutic intervention in the clinic [12]. The shortcomings of these models were hardly failures, nor should they be viewed as such. They actually laid the groundwork for current guidelines used to define a good preclinical model such as high rate of incidence, short latency, molecular and histopathological properties of human disease, and the ability to predict human response to treatment [26].

## 3. Applications to the Clinic: The Good, the Bad, and the Ugly

Direct clinically relevant goals of preclinical studies are to determine preliminary efficacy, toxicity, and pharmacokinetic and safety information of novel drugs in order to support the development of human trials [26,27]. In vitro and in vivo methods can be used to determine if the anticipated therapeutic characteristics of a new drug required for clinical success, such as absorption, disposition, metabolism, elimination, and toxicity, are present [28]. Every approach is individually limited; thus, understanding the benefits and barriers is of utmost importance when designing a study. Utilization of a strategic combination is imperative for successfully translating any findings to the clinic.

Cultured studies were performed for decades to explore the underlying biological mechanisms of cancer development. In vitro studies were used to identify the genetic and epigenetic changes in cancer cells that contribute to tumor initiation [29,30,31,32,33]. In addition, these tools are very useful to predict the response and resistance of cancers to different treatments [28,34,35,36]. Pharmacological high-throughput drug screening is readily applied to identify and evaluate potential therapeutics [29]. Moreover, diagnostically, the use of high-throughput omic analysis showed that molecular signatures of pediatric CNS tumors better predict patient outcome compared to histopathology alone [37,38,39,40,41]. 

Model systems used for in vitro research include mouse or human-derived cell lines and primary cells such as tumor stem cells or neurosphere cultures [29,30,42,43,44]. Cell lines that maintain the genetic perturbations of the primary tumors from which they were derived are ideal models [29]. Approximately 60 cell lines were generated from pediatric brain tumors including ependymomas (EPN), MB, HGG, and AT/RT (extensively reviewed by Xu et al.) [33]. A majority of these lines represent MB, which consists of four genetically distinct subgroups designated Wingless (WNT), Sonic Hedgehog (SHH), Group 3, and Group 4 [41]. In addition to molecular characteristics, prognoses vary between these types of MBs making modeling for specific subgroups a necessity. A newly designed bioinformatic classification tool predicted MB cells DAOY, D425, ONS-76, D283, D341, PFSK-1, D384, D458 to be WNT or SHH subtypes [45]. However, unlike WNT and SHH tumors, many of the lines are *MYC*-amplified like Group 3 MB [41]. Only 12 MB cells line were shown through various analyses to be specifically affiliated with a subgroup [46]. Except for one Group 4, these are all SHH or Group 3 cell lines [46]. A potential explanation for this discrepancy is the requirement of serum for the propagation of many pediatric brain tumor cell lines [33,47,48]. The use of serum to culture primary tumor cells leads to terminal cell differentiation and a homogenous cell population that acquires multiple molecular aberrations, so that, over time, the resulting cell lines differ significantly from the corresponding primary tumors [43]. Even though genetic and phenotypic drift and a lack of heterogeneity occur with cell lines, they are still commonly used in both genetic and pharmacological studies [49]. Cell lines are easy to work with because they are robust, grow rapidly, are easily modified, and can be stored long-term [44]. 

Some of the challenges with cell lines were addressed after the discovery of tumor stem cells when neurosphere cultures from EPN, MB, and astrocytoma pediatric brain tumors were first generated [30,31]. Neurospheres are cultured in the absence of serum and maintain tumor heterogeneity, making them an attractive alternative to serum cultured cells [49,50]. Spheres are heterogeneous and are good candidates for proliferation and differentiation assays despite technical challenges [30,32,42,43,49]. Although studies using sphere lines are a truer reflection of primary tumor behavior compared to cell lines, sphere lines are finicky and rarely maintained by long-term culturing [32,42]. Propagating tumor cells under these conditions select for neural stem-cell-like cells. Many pediatric brain tumors arise from more lineage committed cells; therefore, generating neurosphere cultures from these tumors is not optimal [51,52]. To date, a single Group 4 MB neurosphere cell line (CHLA-01-MED), and a subsequent line from a malignant pleural effusion from the same patient (CHLA-01R-MED) were established and are commercially available (American Type Culture Collection (ATCC): CRL–3021 and CRL-3034, respectively) [49,53]. However new serum-free models, such as the HGG neurosphere lines by Wenger et al., which can be cultured up to 30 passages, continue to be generated [54].

Cell and sphere lines are helpful tools when addressing biological and mechanistic questions. The cost, convenience, and ease of in vitro techniques make them an attractive choice during initial inquiries of novel drugs, particularly for high-throughput testing of therapeutic agents [29]. After positive preliminary results, clinically applicable research of pediatric brain tumors often requires additional evaluation of novel chemotherapeutics and adjunctive therapies in animal models. 

In vivo studies are considered the gold standard in the pharmaceutical industry when addressing drug safety and efficacy questions to support human testing [55]. Many organisms are used in different fields, but the most common host for pediatric brain research is the mouse. Like in vitro, in vivo techniques possess both advantageous and limiting characteristics, all of which require consideration when designing a preclinical trial. Anatomical characteristics of a host animal to cogitate include extracellular matrix molecules, cytokines/growth factors, endothelium, tissue-specific progenitor cells, and immune cells [12]. Depositories like JAX Mice and Services (part of the International Mouse Strain Resource) carry commercially available strains with varying types of immunodeficiency, and some can tolerate clinically relevant levels of radiation. As previously mentioned, the two major variations of murine models used in pediatric brain studies are PDXs and GEMMs. 

**Figure 1:**
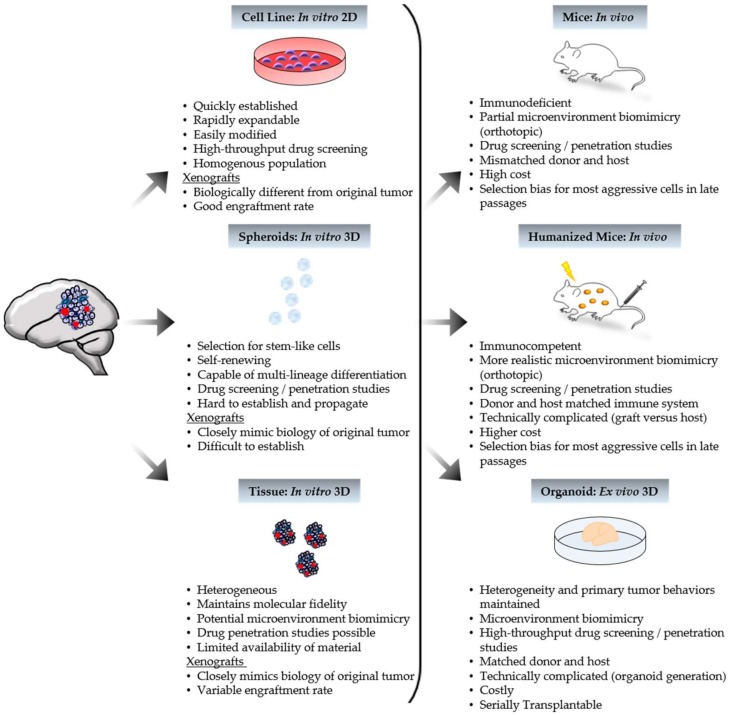
Characteristics of patient-derived model systems. Human tumor tissue can be processed to generate in vitro, ex vivo, and in vivo models. Xenografts can yield from tumor-derived in vitro models, as depicted above, or directly from patient tumor tissue. Represented tumor features include cancer cells (purple), cancer stem cells (blue), and blood vessels (red).

Xenograft modeling refers to the implantation of primary tumor cells/tissue, or tumor-derived cell or sphere lines, classically under the skin (subcutaneous) or, more recently, in the native tumor site (orthotopic) of a host animal (Figure 1). Models generated by implantation of either mouse or human-derived cell lines, are inexpensive and predictable with rapid tumor growth, yet lack tumor heterogeneity and are rarely infiltrative [12,44,49,56]. Syngeneic xenografts, where the host and transplantable materials are genetically and immunologically matched, will contain a realistic microenvironment and intact immune system [12,13,57]. On the other hand, human cell-line models, while lacking both benefits, better represent human biology. Unlike cell lines, transplanting primary sphere lines or tumor tissue allows for maintained heterogeneity [4,16,47,48,51,57,58,59,60,61]. There is also the possibility of a pertinent microenvironment in the low-passage models of primary tumor transplants as tumor stroma may be present [26,44]. For pediatric brain cancer, mainly orthotopic PDXs are applied to drug efficacy comparisons between primary and corresponding metastatic tumors [62]. After a clinical trial, they are also used to identify molecular changes between the pre- and post-treatment tumors of non-responders [12,34].

Of course, there are many caveats to contend with. Results may only be reflective of the individual tumors samples. Multiple passages select the most aggressive cells, depleting heterogeneity [12,44,49,56]. PDXs from patient tumors are limited by the amount of available material and variable engraftment rates (tumor tissue from a patient with poor prognosis is the most efficacious); furthermore, a sufficient cohort comes with high cost and high effort [12,44,49,56]. As with any xenograft, implantation can disrupt the cell–matrix interactions and the blood–brain barrier, and lack tumor initiation [12]. Fortunately, these last three limitations can be bypassed using GEMMs. 

GEMMs are engineered by retroviral, proviral, or chemically induced mutations, including the addition of foreign DNA or transgenes, or by targeted mutations such as truncated or deleted gene knock-outs or amplified gene knock-ins [13,49]. Genes and mutations studied with GEMMs are often associated with a specific human disease and are highly controllable. They may be conventional or conditional to control spatial and temporal expression through the use of systems like Cre-*LoxP* or tetracycline-controlled transcriptional activation (Tet-off/on) [13,49]. GEMMs are often used to investigate individual genetic events in pathogenesis or tumor cell of origin [57]. They can also be designed to imitate rare subgroups. The majority of pediatric brain tumor GEMMs model MBs, mainly representing SHH MB tumors (extensively reviewed by Neumann et al.) [44]. After the link between Gorlin syndrome and aberrant SHH signaling in MB was discovered, many MB GEMMs were generated by modifying SHH signaling genes, such as *Ptch*, *Smo*, or *Sufu*, often in combination with deletion of *Trp53* or cyclin-dependent kinases [63,64,65]. Progenitors of the lower rhombic lip were identified as the cell of origin for WNT MBs after the development of the WNT MB GEMM which is genetically engineered to overexpress *Ctnnb1* and a *Pik3ca* mutation in combination with *Trp53* knock-out [66]. Compared to WNT and SHH, less is known about the drivers of Group 3 and Group 4 MBs, resulting in few models [44]. The GTML (*Glt1-tTA/TRE-MYCN-Luc*) model, a MB GEMM, develops tumors that closely resemble Group 3, but WNT, SHH, and Group 4 can also arise [52,64,67]. There are currently no specific Group 4 MB GEMMs available [44]. Unlike xenografts, GEMMs recapitulate tumor initiation and progression in the presence of the native immune system, blood–brain barrier, cell–matrix interactions, and microenvironment, making them attractive models for targeted therapeutics and drug delivery studies [13]. However, like PDXs, the development and characterization of GEMMs are time-consuming endeavors with a high cost. Tumor penetrance can be incomplete with unpredictable growth [13,44]. GEMM tumors may lack heterogeneity, potentially limiting the ability to recapitulate the genetic complexity seen in human tumors [12,44,49]. Importantly, in the absence of a conserved, phenotypic response to genetic aberrations, the tumorigenesis and drug response seen in GEMMs often differ from humans [13].

Each preclinical pediatric brain tumor model has advantages and pitfalls. Despite many attempts, these models have yet to improve therapeutic options for pediatric patients. Not a single molecular targeting drug was added to a standard treatment protocol for pediatric brain tumors and only one, everolimus, has FDA approval for treatment of nonoperable subependymal giant cell astrocytoma [68]. However, many candidates were identified and are currently under clinical assessment. A promising example is the identification of numerous targets of the SHH pathway in SHH-driven MB. Inhibitors developed against smoothened (SMO) went through phase I and II trials [69]. An interesting finding in the phase II studies showed a wide range of drug efficacy in patients. These results paralleled the discovery of tumor diversity by omic evaluation, which was recapitulated in murine models [70]. Post-clinical research showed a subset of the non-responders had mutations downstream of SMO in the SHH pathway, suggesting a different inhibitor of downstream targets such as zinc finger protein (GLI) may benefit those patients [69]. Research targeting BRD4 (bromodomain-containing protein 4), a BET (bromodomain and extra terminal domain) protein that regulates *GLI* transcription, demonstrated both PDX and GEMM-models of Hedgehog-driven tumors (including SHH MBs and AT/RTs), with genetic perturbations resulting in resistance to SMO inhibitors, responded to treatment with the BRD4 inhibitor JQ1 [71]. This is an excellent example of “from bench to bedside and back again” and exemplifies the need to improve development of preclinical studies as the continued identification of tumor variations and subgroups will require the design of more specific models.

## 4. Promising Progress

A key expectation for a good preclinical model is the ability to predict human response to treatment. Although traditional models are extremely valuable tools, interpreting the results of model studies to predict clinical response may be the biggest challenge to overcome. A good start to addressing this problem is the reevaluation of preclinical strategies. Due to clear differences between mice and humans, ideas to humanize preclinical trials need to be in the forefront of future model designing. 

To diminish time and cost, plus obvious ethical reasons, cultured studies need to be revisited. In vitro research is often mistakenly minimalized in terms of clinical value. However, pharmaceutical companies heavily rely on these methods, and even demonstrated that, at times, product assessment is more translatable with in vitro studies compared to in vivo [27,28,55,72]. In response, the development of some interesting and innovative ideas evolved in recent years. The explosion of multiple omic methodologies resulted in numerous readily available primary tumor datasets including those from the International Cancer Genome Consortium (ICGC), The Cancer Genome Atlas (TCGA), and, for pediatrics, Therapeutically Applicable Research to Generate Effective Treatments (TARGET). These data can be compared to omic studies of human cell lines in order to identify one that best represents a specific cancer subtype [29,73]. Resources are continuously updated and could be used to amplify the generation of new, diverse, and biologically relevant tumor cell lines with potential to enhance the success of drug discovery studies and create more applicable human-derived xenograft models [33,55,74,75].

Another exciting new avenue of in vitro modeling is three-dimensional (3D) growth cultures. Bingle et al. recently determined that 3D prototypes better represent neuroblastoma physiology compared to two-dimensional (2D) cultures [76]. With the use of bioreactor systems, they demonstrated how pertinent biological and physiological conditions, such as shear stress, compound flux, and removal of metabolites, could be studied with 3D cultures [76]. A third promising cell-derived tool for preclinical therapeutic research is the organoid. Organoids are used to study physiological processes in a setting closely resembling endogenous cell organization and organ architecture. An elegant new study by Ogawa et al. demonstrated that a cerebral organoid could recapitulate glioblastoma development via CRISPR/Cas9 (clustered regularly interspaced short palindromic repeats/CRISPR-associated protein 9) oncogene manipulation or transplantation of organoid- or adult-patient-derived glioblastoma cell lines [77]. These are great examples of what can be accomplished. For reasons mentioned above, in vitro studies should always be the first choice when results are equivalent to, or even better than, those obtained using animals. However, for now, most in vitro studies require additional testing before any clinical trial could be considered.

Building better animal models is a continually evolving pursuit as no animal can be a perfect human exemplar. A promising trend, first used to study blood cancers, but recently gaining popularity in solid tumor research, is the humanization of mice. Humanized mice are immunocompromised animals that receive human bone marrow to reconstitute the immune system. Compared to other models, these animals provide a more realistic tumor microenvironment and tumor heterogeneity with the potential for better drug response prediction. Humanized mouse models of diffuse intrinsic pontine glioma (DIPG), HGG, and high-risk MB were utilized to demonstrate that a DNA-damaging reagent, currently in trials to treat lung cancer, called 6-thio-2’deoxyguanosine could cross the blood–brain barrier and selectively enter tumor cells [78]. The downside to working with these animals, like with any murine model, is that they are expensive and technically complicated by the risk of xenogeneic graft-versus-host responses; however, they are a step closer to recapitulating human cancers in animals.

An ideal alternative to mice may be zebrafish (Table 1). The cancer genomes between zebrafish and humans are highly conserved [79]. Patient tumors can be transplanted to form PDXs. Genetically modified models can be generated by adding single or combined mutations [80]. Both approaches could potentially be used for compound toxicity screens, as well as time- and cost-efficient high-throughput in vivo analyses, although the pharmacokinetics of most drugs in zebrafish has yet to be determined [81]. Zebrafish are translucent, allowing direct imaging of tumor behavior with just a microscope [79]. Moreover, a single female can produce up to 200 embryos a day [79]. Successful transplants of mouse-derived ependymoma, glioma, and choroid plexus carcinoma were achieved [81]. More recently, an oligoneural/NB-FOXR2 (NB forkhead box R2) CNS primitive neuroectodermal tumor type-specific model was generated by activating N-RAS in Olig2+/ Sox10+ (oligodendrocyte transcription factor /sex-determining region Y box 10) oligoneural precursor cells of embryonic zebrafish [82]. Mesenchymal glioblastoma and low-grade glioma models were also generated [83]. Whether zebrafish tumors are better representations of human tumors than mice is still unknown, but early studies indicate they will be valuable tools for future research.

## 5. Concluding Remarks

In order to improve the clinical relevancy of preclinical research, we must acknowledge the major hurdles impeding our progress. Preclinical versus clinic studies and endpoints do not resemble each other. Slowing of tumor progression as a measurement of disease response to treatment is defined in the clinic as complete response, partial response, and overall increase in survival versus in the lab as tumor growth inhibition and tumor growth delay. The latter criteria do not directly correlate to an overall increase in survival. The discrepancy between these definitions of success and failure impede both discovery and extensive testing of prospective therapeutics. The challenge to bridge basic science and clinical communication is still in front of us. Multidisciplinary collaborations are being developed worldwide as a potential solution, and time will tell if they are enough to maximize preclinical–clinical synergy. Then, there are the funding limitations. As discussed in this review, no single model can currently replicate the development, diversity, or drug responsiveness of a human tumor; furthermore, a dismal percentage of compounds identified by preclinical cancer research become beneficial therapeutics in the clinic. Therefore, a question deserving serious consideration is whether investing more upfront for preclinical research would lead to better model design and improve drug discovery. Regardless of the solution, increased efficiency in the lab is necessary to ultimately decrease the current number of unsuccessful and expensive human trials.

## Figures and Tables

**Table 1:** Zebrafish in cancer modeling.

	Benefits			Limitations
**Translational relevance: similarities between humans and zebrafish**	Largely conserved development and signaling pathways	Function of innate and adaptive immune cells is highly conserved	Over 80% of human disease-related genes present	Whole-genome duplication (more than one ortholog for some human genes) may interfere with genetic studies
**Xenograft models **[79,81,84]	Can be generated from human, mouse, or zebrafish tumors	No rejection due to immature adaptive immune system in larvae	Recapitulates parental tumor behaviors including proliferation, survival, invasion, and dissemination	Molecular interactions between transplanted human or mouse tumors and zebrafish cells unclear
**Genetically engineered zebrafish models (GEZMs) **[82,85,86]	Easy genetic manipulation—injection into one-cell-stage larvae possible	Fast development	Comparable histology to human cancers	Tumor initiation and progression studies hindered by lack of a functional adaptive immune system in early-stage models
**Drug studies**	Easy, cost-effective, and high scalability	Ease of imaging and high-throughput screening with transparent larvae	High degree of conservation of metabolic enzymes between human and zebrafish larvae	Pharmacokinetic processes still unclear
